# Longitudinal changes in HIV DNA in HIV controllers: what do they mean?

**DOI:** 10.1002/jia2.25254

**Published:** 2019-02-15

**Authors:** Denise C Hsu, John W Mellors

**Affiliations:** ^1^ Department of Retrovirology AFRIMS Bangkok Thailand; ^2^ US Military HIV Research Program Walter Reed Army Institute of Research Silver Spring MD USA; ^3^ Henry M. Jackson Foundation for the Advancement of Military Medicine Bethesda MD USA; ^4^ Division of Infectious Diseases Department of Medicine University of Pittsburgh School of Medicine Pittsburgh PA USA

**Keywords:** HIV controllers, HIV reservoir, HIV DNA, HIV cure

The existence of the latent HIV reservoir is a major barrier to HIV cure 1. Understanding what maintains the latent reservoir and how latently infected cells are eliminated will help advance HIV cure efforts.

For the majority of individuals living with HIV, levels of plasma genomic HIV RNA and cellular HIV DNA and mRNA rise rapidly and peak within the first month following infection, then plateau 2. After antiretroviral therapy (ART) is initiated, HIV RNA levels decline rapidly (by 10,000‐fold) and can become undetectable by clinical assays. HIV DNA levels also decline, but less impressively (10‐ to 20‐fold) 2, 3, 4. Modelling demonstrates that HIV DNA decay kinetics fit a 3‐slope curve over three time intervals: zero to seven months, eight to thirty‐two months and >32 months, with slopes of decay of −0.131, −0.016 and −0.0021 log_10_ copies/10^6^ peripheral blood mononuclear cells (PBMC)/month over the respective time periods 5.

HIV controllers are a small group (approximately 1%) of individuals who are able to control viral replication without ART to very low or undetectable levels of HIV‐1 RNA by clinical assays for long periods of time 6. The mechanisms involved in their viral control likely include a combination of both viral and host factors 7. The longitudinal kinetics of infected cell decay (i.e. of HIV DNA) in HIV controllers are less well described.

In their recent article in the *Journal of the International AIDS Society*, Avettand‐Fenoel *et al*. described HIV DNA kinetics in 202 HIV controllers (defined as having HIV RNA <400 copies/mL without ART) from the ANRS‐CODEX cohort 8. The median HIV DNA was 1.5 log_10_copies/10^6^ PBMC, which is much lower than the 3.3 log_10_ copies/10^6^ PBMC in ART‐naïve individuals during primary HIV infection in the ANRS‐PRIMO cohort 9, but similar to the predicted 1.6 log_10_ copies/10^6^ PBMCs after five years of uninterrupted ART in individuals started on ART within 15 days after HIV infection in the same cohort 5. Mathematical modelling of HIV DNA dynamics in the ANRS‐CODEX HIV controller cohort revealed a significant decline in 46% of participants. HLA‐B*27/B*57 alleles and lower levels of plasma HIV RNA and HIV DNA at the entry visit into the cohort were independently associated with HIV DNA decline.

The authors postulated that intrinsic resistance of host cells to HIV infection, lower levels of immune activation leading to fewer potential target cells, lower residual HIV replication and a shorter half‐life of infected cells may all have contributed to the observed decline in HIV DNA levels. By contrast, non‐HLA‐B*27/B*57 alleles or persistent HIV RNA ≥1 log_10_ copies/mL during follow‐up were associated with increases in HIV DNA over time in the same controller cohort. Characterization of the HIV proviruses by DNA sequencing and analyses of clonal expansion of infected cells in the controllers could provide additional insight into the mechanisms behind the divergent HIV DNA dynamics that were observed 10. Such analyses would differentiate clonal expansion of cells with identical proviruses from ongoing cycles of infection as the cause of rising HIV DNA levels.

Although a decline in HIV DNA levels in HIV controllers is intriguing, it does not necessarily imply a reduction in the latent but replication‐competent (intact) proviral reservoir that can produce infectious virus and lead to viral rebound. This is because unintegrated linear and episomal DNAs, in addition to integrated proviruses, are also detected by total HIV DNA quantification, neither of which can produce infectious virus. HIV controllers have also been found to have a higher proportion of unintegrated DNA to total HIV DNA when compared to both treated and untreated non‐controllers 11. In addition, the vast majority of integrated proviral DNA is defective and cannot lead to the production of infectious virions 12. Though assays designed to quantify replication‐competent provirus may be able to provide a more accurate estimate of the latent reservoir, these assays are labour intensive and may lack the dynamic range to detect small fluctuations 13. Newer PCR‐based assays of intact provirus should provide further insight into whether the changes observed in HIV DNA levels in HIV controllers in the ANRS‐CODEX cohort parallel those of the intact reservoir.

## Competing interests

JWM is a consultant to Gilead Sciences and Merck, and has share options in Co‐Crystal Pharmaceuticals, Inc. DCH has no conflict of interest to declare.

## Authors’ contributions

DCH and JWM drafted the letter.
